# 9-Year Serial CMR Monitoring of Cardiac AL Amyloidosis Regression After Teclistamab

**DOI:** 10.1016/j.jaccas.2026.109553

**Published:** 2026-08-27

**Authors:** Valentina Silvestri, Manuel Toledano, Bénédicte Hivert, Emmanuelle Bourgeois, Benjamin Carpentier, Jean Giraud, Alexandre Willaume, François Delelis, Laurent Pascal

**Affiliations:** a Department of Radiology and Cardiovascular Imaging, GHICL–Groupement des Hôpitaux de l’Institut Catholique de Lille, Lille, France; b Department of Hematology, GHICL–Groupement des Hôpitaux de l'Institut Catholique de Lille–Hôpital Saint-Vincent-de-Paul, Lille, France

**Keywords:** imaging, MR sequences, treatment

## Abstract

**Background:**

Cardiac amyloid light-chain (AL) amyloidosis carries a poor prognosis and is considered largely irreversible once symptomatic. Long-term serial cardiovascular magnetic resonance (CMR) data following B-cell maturation antigen-directed therapy are lacking.

**Case Summary:**

A 64-year-old man with biopsy-proven cardiac AL amyloidosis achieved sustained hematologic complete response after teclistamab following failure of 4 prior lines including daratumumab. Eleven CMR examinations over 9 years documented a biphasic trajectory: progressive infiltration through 2020 (left ventricular mass 186 g, wall thickness 18 mm, global longitudinal strain −4.8%), followed by sustained structural and functional regression through 2025 (left ventricular mass 127 g, wall thickness 13 mm, global longitudinal strain −13.7%).

**Discussion:**

This represents the longest serial CMR follow-up in cardiac AL amyloidosis reported to date, demonstrating that cine-derived parameters reliably track cardiac regression in real-world conditions. Native T1 normalized while extracellular volume fraction (ECV) paradoxically increased, suggesting persistent fibril burden beyond hematologic clearance.

**Take-Home Message:**

Serial CMR with cine-derived parameters provides robust longitudinal monitoring of cardiac AL amyloidosis regression following novel plasma cell–directed therapy, even without systematic contrast enhancement.

**Visual Summary:**
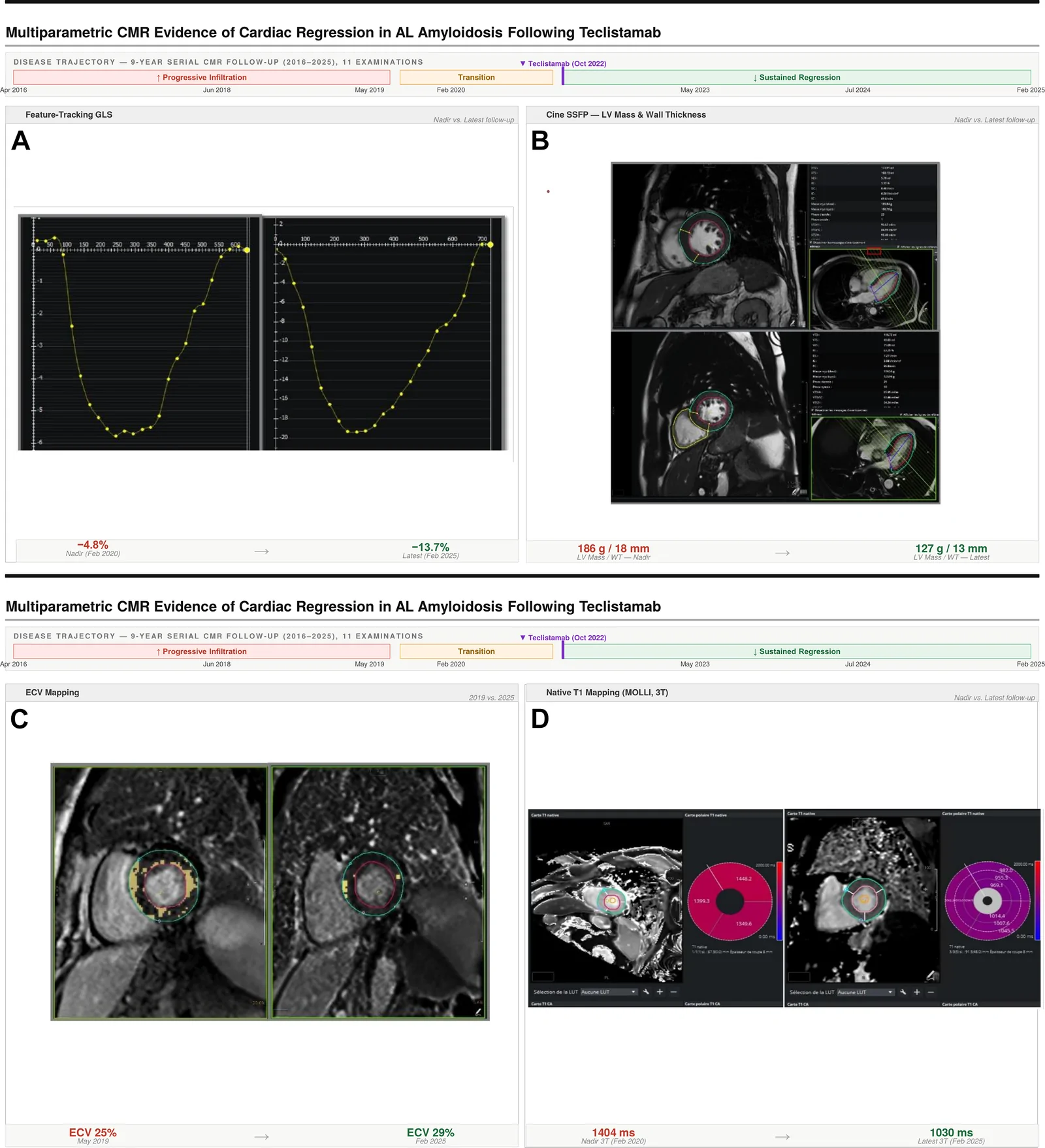

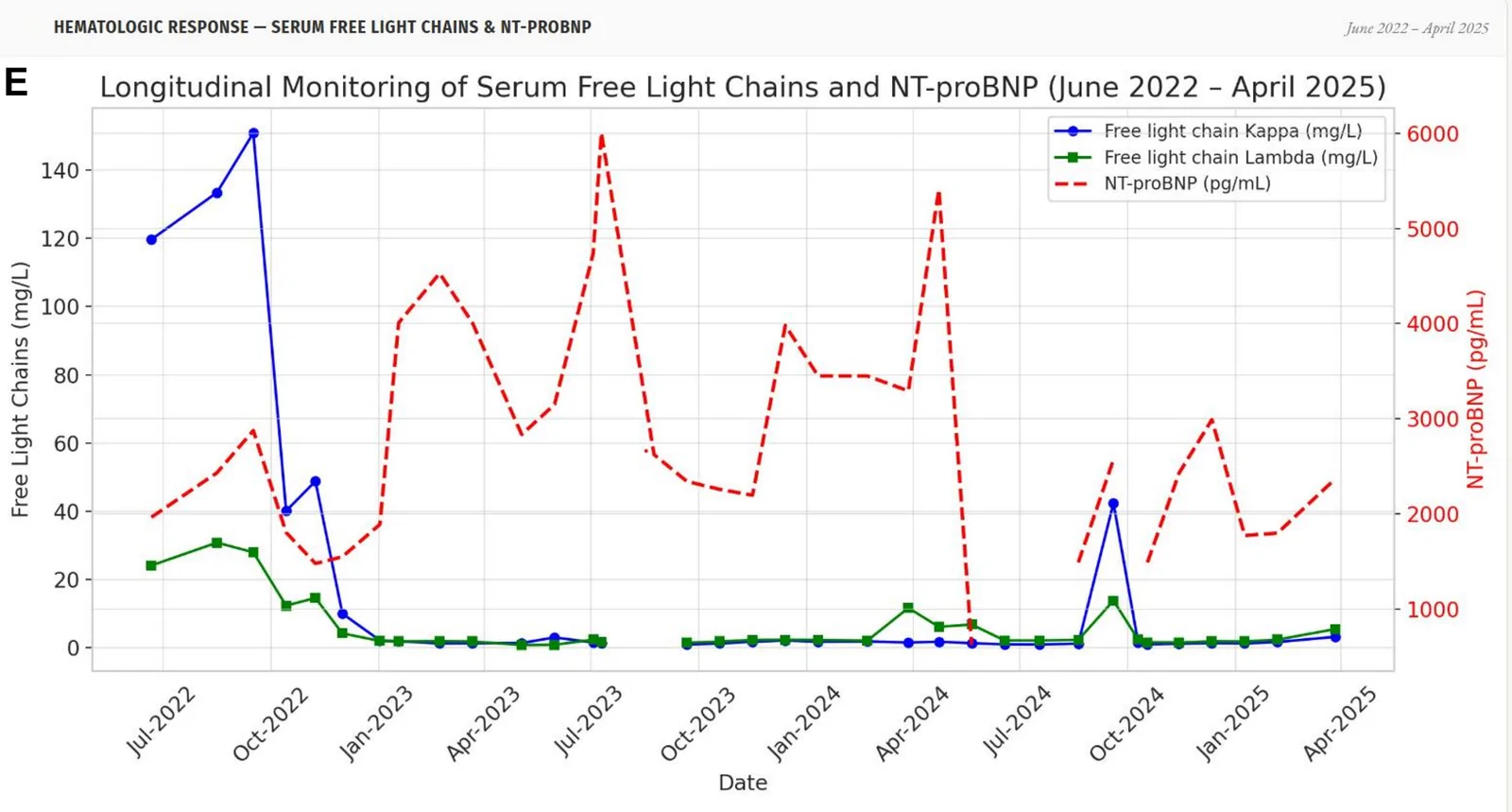
Multiparametric CMR Evidence of Cardiac Regression in AL Amyloidosis Following Teclistamab (A) Feature-tracking GLS curves at nadir (February 2020, GLS −4.8%) and latest follow-up (February 2025, GLS −13.7%), illustrating recovery of longitudinal myocardial contractile function. (B) Short-axis cine SSFP at nadir (LV mass 186 g, WT 18 mm) and latest follow-up (LV mass 127 g, WT 13 mm), demonstrating progressive regression of LV hypertrophy. (C) ECV color maps at nadir (25%, May 2019) and latest follow-up (29%, February 2025). Persistent ECV elevation despite structural regression is consistent with residual amyloid fibril burden after hematologic clearance. (D) Native T1 maps (MOLLI, 3T) at nadir (1,404 ms, February 2020) and latest follow-up (1,030 ms, February 2025), confirming normalization of myocardial T1 signal. (E) Serum free light chains and NT-proBNP trajectories following teclistamab initiation (October 2022), demonstrating sustained hematologic complete response. NT-proBNP fluctuations reflect dialysis-related fluid status. AL = amyloid light-chain; CMR = cardiovascular magnetic resonance; ECV = extracellular volume fraction; GLS = global longitudinal strain; LV = left ventricle; MOLLI = modified look-locker inversion recovery; NT-proBNP = N-terminal pro-B-type natriuretic peptide; SSFP = steady-state free precession; WT = wall thickness.

## History of Presentation

A 64-year-old man presented in April 2016 with nephrotic syndrome and fatigue. Workup revealed kappa light-chain amyloid light-chain (AL) amyloidosis with bone marrow plasma cell infiltration of 15% and biopsy-proven cardiac involvement. Initial cardiac magnetic resonance (CMR) demonstrated mild left ventricular (LV) hypertrophy (LV mass 139 g, wall thickness 13 mm) with preserved left ventricular ejection fraction (66%) and mildly impaired global longitudinal strain (GLS) (−14.2%).

**Take-Home Messages**

• Serial CMR with cine-derived parameters (LV mass, wall thickness, feature-tracking GLS) provides robust longitudinal monitoring of cardiac AL amyloidosis regression following novel plasma cell–directed therapy, even without systematic contrast enhancement.
• Hematologic complete response after B-cell maturation antigen-directed therapy can be associated with sustained and measurable structural cardiac regression over years, as documented by the longest serial CMR follow-up reported in this setting.

## Past Medical History

End-stage renal disease requiring peritoneal dialysis (initiated early 2022). Previous treatment lines: CyBorD (first line, complete hematologic response), lenalidomide-based (second line), pomalidomide-based (third line), daratumumab-based (fourth line, refractory). No significant cardiovascular history before AL amyloidosis diagnosis.

## Differential Diagnosis

The differential diagnosis of cardiac hypertrophy in this context included hypertensive heart disease, hypertrophic cardiomyopathy, and transthyretin amyloidosis. Biopsy confirmation of AL amyloidosis with kappa light-chain restriction, combined with characteristic CMR features (diffuse subendocardial late gadolinium enhancement, elevated native T1), established the diagnosis of cardiac AL amyloidosis.

## Investigations

Eleven CMR examinations were performed between 2016 and 2025 at 1.5 T or 3.0 T, with gadolinium in 6 of 11 studies. Peak cardiac involvement was documented in February 2020: LV mass 186 g, maximal wall thickness 18 mm, GLS −4.8%, native T1 1,404 ms (3T). Serum free light-chain and N-terminal pro–B-type natriuretic peptide trajectories are shown in the Visual Summary (panel E). At peak disease, the patient met criteria for Mayo Stage IIIa cardiac amyloidosis.^1^^,^^2^

## Management

Teclistamab (B-cell maturation antigen×CD3 bispecific antibody) was initiated in October 2022 as fifth-line therapy. Hematologic complete response (difference between involved and uninvolved free light chains near 0 mg/L) was achieved within 6 months and sustained through the end of follow-up in February 2025.^3^, ^4^, ^5^

## Outcome and Follow-Up

Following teclistamab initiation, serial CMR demonstrated progressive and sustained cardiac structural and functional regression: LV mass decreased from 186 g to 127 g (−32%), maximal wall thickness from 18 mm to 13 mm, and GLS improved from −4.8% to −13.7%. LV ejection fraction remained preserved throughout (59%-67%). Native T1 normalized (1,404 ms → 1,030 ms at 3.0-T), whereas extracellular volume fraction showed a paradoxical slight increase (25% → 29%), consistent with residual amyloid fibril burden persisting beyond hematologic clearance. N-terminal pro–B-type natriuretic peptide fluctuations reflected dialysis-related fluid status rather than cardiac progression. The full longitudinal CMR dataset is presented in Table 1 and the Visual Summary.

**Table 1:** Serial Cardiac Magnetic Resonance Parameters Over 9 Years

Date	Gd	LVEF (%)	LVEDV (mL)	LV Mass (g)	Max WT (mm)	GLS (%)	Native T1 (ms)	ECV (%)
Apr 2016	No	66	147	139	13	−14.2	—	—
Jun 2018	No	63	142	140	15	−11.8	—	—
May 2019	Yes	64	174	186	16	−7.3	—	25
Feb 2020	No	61	135	179	18	−4.8	1,404 (3T)	—
May 2022	No	67	134	138	15	−9.1	—	—
Nov 2022^a^	Yes	67	160	169	16	−7.6	—	—
May 2023	Yes	67	144	125	14	−10.2	—	—
Nov 2023	Yes	63	119	122	15	−11.4	—	—
Jul 2024	Yes	67	162	129	13	−12.8	—	—
Feb 2025	Yes	59	141	127	13	−13.7	1,030 (3T)	29

T1 values not directly comparable across field strengths (3.0 T vs 1.5 T).

ECV = extracellular volume fraction; Gd = gadolinium contrast; GLS = global longitudinal strain; LV = left ventricle; LVEDV = left ventricular end-diastolic volume; LVEF = left ventricular ejection fraction; WT = wall thickness.

a Pre-teclistamab baseline CMR (November 2022). —indicates parameter not available at that examination.

## Discussion

To our knowledge, this case represents the longest serial CMR follow-up in cardiac AL amyloidosis reported to date—11 examinations over 9 years—with sustained cardiac regression documented after B-cell maturation antigen-directed bispecific antibody therapy.^3^, ^4^, ^5^ The biphasic trajectory observed (progressive infiltration 2016-2020, sustained regression 2022-2025) is consistent with the temporal relationship between hematologic response and organ recovery described in AL amyloidosis.^2^

Cine-derived parameters—LV mass, wall thickness, and feature-tracking GLS—proved robust across all 11 examinations despite real-world variability in field strength and contrast availability.^6^^,^^7^ These findings support their use as the primary imaging endpoints for cardiac response monitoring. Native T1 normalization provided complementary structural evidence, whereas the paradoxical extracellular volume fraction increase highlights the dissociation between hematologic clearance and fibril resorption—a finding of independent scientific interest that warrants prospective investigation.^8^

This single-patient observation cannot establish causality, and the real-world imaging heterogeneity (1.5- vs 3.0-T, variable contrast protocols) represents a limitation. Prospective multicenter studies are needed to validate standardized, contrast-optional CMR protocols for cardiac response assessment in patients receiving novel plasma cell–directed therapies.^7^^,^^9^

## Conclusions

This case provides the longest serial CMR documentation of cardiac regression in AL amyloidosis to date. Based on this single-patient observation, cine-derived parameters proved robust and sufficient for longitudinal monitoring despite real-world imaging heterogeneity. Prospective studies in larger cohorts are needed to validate a standardized, contrast-optional CMR protocol for cardiac response assessment.

## Funding Support and Author Disclosures

The authors have reported that they have no relationships relevant to the contents of this paper to disclose.
